# A comprehensive model for tRNA methylation modification studies

**DOI:** 10.1002/mco2.402

**Published:** 2023-10-21

**Authors:** Qi Weng, Feng Zhang, Quan Zheng

**Affiliations:** ^1^ Quzhou People's Hospital The Quzhou Affiliated Hospital of Wenzhou Medical University Quzhou China; ^2^ Department of Pharmacy Quzhou People's Hospital The Quzhou Affiliated Hospital of Wenzhou Medical University Quzhou China; ^3^ Core Facility, Quzhou People's Hospital The Quzhou Affiliated Hospital of Wenzhou Medical University Quzhou China

## Abstract

The molecular mechanism of N^7^‐methylguanosine (m^7^G)‐tRNA modification mediated by the methyltransferase like 1‐WD repeat domain 4 (METTL1–WDR4). Route A indicated that with WDR4 as the scaffold, METTL1 catalyzed G46 methylation modification after binding of the tRNA variable loop via its αC and α6 helices, as reported by Richard I. Gregory's group. Route B showed the METTL1–WDR4‐mediated tRNA methylation modification model and the effect of binding of the *S*‐adenosylmethionine on the N‐terminus of METTL1, as reported by Yunsun Nam's group.

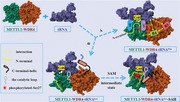

1

Two new studies published back‐to‐back in *Nature* revealed the crystal structure of the methyltransferase like 1‐WD repeat domain 4 (METTL1–WDR4) and the cryo‐electron microscopy (cryo‐EM) structure of METTL1–WDR4–tRNA, and elaborated the molecular mechanism of N^7^‐methylguanosine (m^7^G)‐tRNA modification mediated by METTL1–WDR4.^1^, ^2^ These new breakthroughs lay an important foundation for future researches on the mechanism of METTL1–WDR4‐mediated m^7^G‐tRNA modification in cancer occurrence and provide new ideas to design and develop METTL1–WDR4 methyltransferase inhibitors as potential novel anticancer medications.

In epitranscriptomics research, RNA modification is an important posttranscriptional regulator of gene expression program, which affects the growth and development of various eukaryotes.^3^ The folding, stability, and function of tRNA are affected by extensive posttranscriptional modifications.^4^ In mammals, m^7^G at position 46 of tRNA (m^7^G46) is one of the most prevalent tRNA modifications, catalyzed by the METTL1–WDR4 methyltransferase complex, which can affect tumor cell genesis and progression by regulating the level of steady‐state tRNAs.^5^ The catalytic enzyme METTL1 plays a catalytic role in the METTL1–WDR4 complex, whereas WDR4 lacks catalytic activity and serves as the complex's stable scaffold.^5^ At present, METTL1–WDR4 has been considered as a potential new target for cancer treatment.^1^ However, the paucity of structural data of the METTL1–WDR4 complex and its mechanism of tRNA methylation and modification has posed great challenges for the design of METTL1–WDR4‐targeted inhibitors and the development of novel antitumor drugs.

The latest research paper published in *Nature* by Richard I. Gregory's group revealed mechanism by which METTL1–WDR4 specifically recognized and combined tRNA substrates, emphasized the critical function of METTL1 N‐terminus in tRNA modification, and expounded the effect of METTL1 N‐terminus phosphorylation on methyltransferase activity.^1^ By comparing the cryo‐EM structures of METTL1–WDR4–tRNA^Phe^ and METTL1–WDR4–tRNA^Val^ complexes, the authors observed that the overall structure of both ternary complexes was similar to a sail‐boat‐like arrangement. Furthermore, by superimposing the structures of METTL1–WDR4–tRNA^Phe^ and METTL1–WDR4, the authors discovered that the only region of METTL1–WDR4 that underwent a structural rearrangement after binding to tRNA was the αC loop, which indicated that αC loop formation of αC helix promoted the recognition of tRNA by METTL1–WDR4. In addition, the authors found that WDR4 acted as a scaffold during METTL1–WDR4 binding to tRNAs. B4 of WDR4 not only mediated the interaction with METTL1 but also participated in the binding of tRNAs to B3, suggesting that B3–B4 was essential for the methylation activity of METTL1–WDR4. In the presence of METTL1–WDR4, tRNA would partially unravel and bend to bring its variable loop closer to the METTL1 catalytic pocket, where it would flip its G46 site into the catalytic pocket for methylation modification. In short, using WDR4 as the stable scaffold, METTL1 bound to the tRNA variable loop via αC and α6 helices, catalyzed its binding to the flipped G46 site, thereby achieving m^7^G46 modification of tRNA (Figure 1).

**FIGURE 1:**
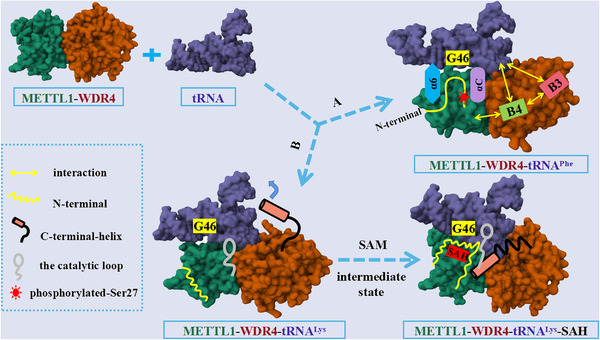
The molecular mechanism of N^7^‐methylguanosine (m^7^G)‐tRNA modification mediated by the methyltransferase like 1‐WD repeat domain 4 (METTL1–WDR4). Route A indicated that with WDR4 as the scaffold, METTL1 catalyzed G46 methylation modification after binding of the tRNA variable loop via its αC and α6 helices, as reported by Richard I. Gregory's group. Route B showed the METTL1–WDR4‐mediated tRNA methylation modification model and the effect of binding of the *S*‐adenosylmethionine (SAM) on the N‐terminus of METTL1, as reported by Yunsun Nam's group.

Notably, the authors verified that its N‐terminal portion (residues 1−33) was disordered by expressing isotope‐labeled METTL1. Then, the authors found that residues 18−27 were related to the central region of METTL1 through nuclear magnetic resonance (NMR) titration experiments. Truncation of the METTL1 N‐terminus and mutagenesis of the METTL1 α2 helix showed that residues 16−27 were crucial to METTL1 activity. In addition, after NMR analysis of the S27D mutant at the N‐terminal of METTL1, the authors pointed out that the phosphorylation of S27 would cause steric hindrance and would introduce negative charges to repel tRNA as well as destroy the catalytic center of METTL1, thus inhibiting its methylation activity.^1^

At the same time, the latest research paper published in *Nature* by Yunsun Nam's group clarified how METTL1 and WDR4 cooperated with each other to recognize tRNA substrates and catalyze m^7^G46 modification.^2^ First, through in vitro methylation determination and electrophoretic mobility shift assays, the authors confirmed that METTL1 could better combine with tRNA^Lys^ and catalyze its methylation modification only when it formed METTL1–WDR4 complex with WDR4. In addition, the authors found that the molecular interactions between WDR4 and METTL1 as well as between *S*‐adenosylmethionine (SAM) or *S*‐adenosylhomocysteine (SAH) and METTL1 were critical to the activity of METTL1–WDR4 complexes, through the crystal structure and site‐directed mutagenesis studies of METTL1–SAM, METTL1–SAH, and METTL1–WDR4 complexes. Then, the cryo‐EM structure of METTL1–WDR4–tRNA revealed that METTL1–WDR4 complex specifically bound tRNA through shape and charge complementary form. Meanwhile, by comparing the cryo‐EM structures of METTL1–WDR4–tRNA, METTL1–WDR4–tRNA–SAM, and METTL1–WDR4–tRNA–SAH, the authors observed that the conformation of the methyl donor cofactor SAM binding state was an intermediate state, whereas there was a significant difference in the conformation of the apo and cofactor demethylation product SAH binding states. The C‐terminal helix of WDR4 becomes more stable and orderly in the SAH binding state, which is crucial to the formation of optimal enzyme activity. However, the helix is flexible in the apo and SAM binding states. Besides, the truncation and induced mutation analysis of the C‐terminal helix of WDR4 showed that its truncation and induced mutation would reduce the methylation ability of the complex. Using the structural and biochemical experimental data, the authors found that the truncation of the METTL1 N‐terminus or the phosphorylation of S27 could inhibit the methylation activity of METTL1–WDR4. Moreover, they observed that the N‐terminal of METTL1 was disordered without cofactor binding. However, after binding with SAH, the N‐terminal of METTL1 would cross the narrow channel between SAH and the D arm of tRNA orderly (Figure 1). The N‐terminal of METTL1 regulated the methylation activity of m^7^G46 by coordinating the binding of tRNA and SAM with METTL1–WDR4 and corresponding conformations changes, while the phosphorylation of S27 interfered with their interaction through steric hindrance and charge repulsion.^2^

In summary, two studies have jointly expounded the molecular mechanism of how METTL1 and WDR4 cooperated to recognize tRNA and catalyze m^7^G46 modification. Richard I. Gregory's group mainly focused on the key role of truncation or mutation of specific residues on the N‐terminal of METTL1 in tRNA modification, whereas Yunsun Nam's group concentrated on the effect of cofactor binding on the conformation change and methylation activity of METTL1–WDR4. The discovery of the molecular mechanism will provide a necessary basis for analyzing the pathogenesis of related diseases.^2^ Currently, METTL1–WDR4 has been considered as a promising new target for cancer therapy, but its biological function has not been thoroughly studied, and no related inhibitors have been reported so far.^1^ Therefore, to further elucidate how METTL1–WDR4 recognizes and modifies its RNA substrate, while promoting the clinical transformation of research in this field, future work might be carried out in the following areas: (1) based on the METTL1–WDR4‐mediated m^7^G‐tRNA modification model, we can extensively study the methylation modification of other tRNAs catalyzed by METTL1–WDR4 and explore whether METTL1–WDR4 participates in regulating other RNA modifications; (2) to gain a deeper understanding of the relationship between the dysregulation of m^7^G tRNA modification and disease occurrence, further researches should be conducted in animal models to investigate the mechanism of METTL1–WDR4‐mediated m^7^G tRNA modification in cancer development; (3) based on the new finding that the N‐terminus of METTL1 and the C‐terminus of WDR4 play important roles in the methylation activity of METTL1–WDR4 in this study, it is possible to design and develop METTL1–WDR4 methyltransferase inhibitors targeting these two regions, which will provide new directions for the early diagnosis and treatment of cancer.

## AUTHOR CONTRIBUTION

Q. Z. conceptualized the manuscript. Q. W. and Q. Z. wrote the manuscript and prepared the figure, and F. Z. proofread and edited the manuscript. All authors have read and approved the article.

## CONFLICT OF INTEREST STATEMENT

The authors declare no conflict of interest.

## Data Availability

Not applicable.

## References

[1] Li J , Wang L , Hahn Q , et al. Structural basis of regulated m^7^G tRNA modification by METTL1‐WDR4. Nature. 2023;613:391‐397. 36599985 10.1038/s41586-022-05566-4 PMC11179147

[2] Ruiz‐Arroyo VM , Raj R , Babu K , et al. Structures and mechanisms of tRNA methylation by METTL1‐WDR4. Nature. 2023;613:383‐390. 36599982 10.1038/s41586-022-05565-5 PMC9930641

[3] Frye M , Harada BT , Behm M , et al. RNA modifications modulate gene expression during development. Science. 2018;361:1346‐1349. 30262497 10.1126/science.aau1646 PMC6436390

[4] Han H , Yang C , Ma J , et al. N^7^‐methylguanosine tRNA modification promotes esophageal squamous cell carcinoma tumorigenesis via the RPTOR/ULK1/autophagy axis. Nat Commun. 2022;13:1478. 35304469 10.1038/s41467-022-29125-7 PMC8933395

[5] Chen J , Li K , Chen J , et al. Aberrant translation regulated by METTL1/WDR4‐ mediated tRNA N^7^‐methylguanosine modification drives head and neck squamous cell carcinoma progression. Cancer Commun. 2022;42:223‐244. 10.1002/cac2.12273 PMC8923133 35179319

